# Itacitinib plus calcineurin inhibitor–based therapy for prophylaxis of graft-versus-host disease: GRAVITAS-119 results

**DOI:** 10.21203/rs.3.rs-9915719/v1

**Published:** 2026-06-11

**Authors:** Nirav Shah, Hannah Choe, nancy Hardy, Patrice Chevallier, Marie Therese Rubio, Mark Schroeder, Carlos Solano, Amelia Langston, Patrick Stiff, Kevin Hou, Michael Arbushites, Michael Pratta, Rodica Morariu-Zamfir, Miguel Angel Perales

**Affiliations:** Medical College of Wisconsin; James Cancer Hospital, The Ohio State University Comprehensive Cancer Center; UMD; CHU Hotel Dieu; Hopital Brabois; Washington University School of Medicine in Saint Louis; Hospital Clínico Universitario de Valencia. Universitat de Valencia. Instituto de Investigación Sanitaria INCLIVA; Emory University School of Medicine; loyola University Medical Center; Incyte Corporation; Incyte Corporation; Incyte Corporation; Incyte Corporation; Memorial Sloan Kettering Cancer Center

**Keywords:** itacitinib, prophylaxis, reduced-intensity conditioning, cyclophosphamide

## Abstract

GRAVITAS-119 (NCT03320642), a single-arm, open-label, phase 1, proof-of-concept study, assessed addition of itacitinib to standard-of-care graft-versus-host disease (GVHD) prophylaxis regimens in reduced-intensity conditioning peripheral blood allogeneic hematopoietic stem cell transplant. Day 28 hematologic recovery (percentage of patients demonstrating neutrophil recovery and platelet recovery) was the primary endpoint. Of 84 enrolled patients, 41 received itacitinib plus tacrolimus/methotrexate (nine with antithymocyte globulin [ATG]), 24 received itacitinib plus cyclosporine A/mycophenolate mofetil (16 with ATG), and 19 received itacitinib plus post-transplant cyclophosphamide/tacrolimus. Hematologic recovery was achieved by 51/55 (92.7%) evaluable patients (neutrophil recovery, 98.7%; platelet recovery, 94.6%). Estimated 1-year GVHD-free, relapse-free survival was 39.7%; estimated 1-year overall survival was 78.3%. Cumulative incidences for grade 2–4 and grade 3–4 acute GVHD at Day 180 were 13.2% and 4.8%, respectively; cumulative incidence of chronic GVHD at 1 year was 37.8%. Most common grade ≥ 3 adverse events were cytopenias; grade ≥ 3 infections occurred in 23 (27.4%) patients. Most patients receiving itacitinib plus standard-of-care GVHD prophylaxis achieved hematologic recovery, with any-grade GVHD and infection rates similar to previous reports with standard-of-care prophylaxis. Addition of itacitinib to GVHD prophylaxis permits timely hematologic recovery and does not increase GVHD or infection rates, although unmet need still remains for improved regimens.

## INTRODUCTION

Allogeneic hematopoietic stem cell transplant (allo-HSCT) offers potentially curative therapy for patients with bone marrow and immune disorders. However, serious complications following allo-HSCT include opportunistic infections, medication-associated morbidities, and graft-versus-host disease (GVHD) [1–3]. Acute GVHD (aGVHD) occurs in approximately 30%-60% of allo-HSCT recipients and causes substantial mortality and morbidity. Chronic GVHD (cGVHD) occurs in 30%-50% of allo-HSCT recipients and is a major cause of morbidity and non-relapse mortality [3–7].

Standard-of-care GVHD prophylaxis includes calcineurin inhibitors (CNIs) such as cyclosporine A (CsA) or tacrolimus plus methotrexate when administered after myeloablative conditioning and allo-HSCT [1, 8]. For older patients, CNI-based prophylaxis regimens with reduced-intensity conditioning (RIC) are preferred [9]. Addition of antithymocyte globulin (ATG) to standard prophylactic regimens may further decrease GVHD rates in both matched-related and -unrelated donor transplants and is recommended by the European Society for Blood and Marrow Transplantation, albeit without a survival benefit [10–13]. Abatacept provides co-stimulatory blockade of T-cell activation and effectively reduces risk of severe grade 3–4 aGVHD when added to standard CNI-based prophylaxis plus methotrexate; however, a four-dose regimen had no effect on the later incidence of cGVHD [14]. Finally, results from the BMT CTN 1703 study show improved GVHD-free, relapse-free survival (GRFS) for those receiving post-transplant cyclophosphamide (PTCy) plus tacrolimus and mycophenolate mofetil (MMF) versus tacrolimus/methotrexate following RIC [15]. However, variability exists across clinical practices, and many patients ultimately develop GVHD despite receiving prophylactic regimens [16, 17].

Janus kinase (JAK) inhibition is an efficacious and tolerable therapeutic approach for treatment of GVHD, as demonstrated with the JAK1/JAK2 inhibitor ruxolitinib in the pivotal REACH1, REACH2, and REACH3 studies that led to ruxolitinib approval for treatment of steroid-refractory aGVHD and cGVHD after failure of systemic therapy [18–21]. The selective JAK1 inhibitor itacitinib was evaluated in a phase 1 study in patients with steroid-naive or steroid-refractory aGVHD based on the hypothesis that itacitinib might provide additional benefit versus JAK1/JAK2 inhibition. Although itacitinib was well tolerated and showed preliminary efficacy, the placebo-controlled GRAVITAS-301 study did not meet its primary efficacy endpoint of Day 28 response rate [22, 23].

We report results from the GRAVITAS-119 study, designed to assess the safety, efficacy, and correlative results of the addition of itacitinib to GVHD prophylaxis regimens.

## METHODS

### Study Design

This single-arm, open-label, proof-of-concept study (GRAVITAS-119; NCT03320642) was conducted at 16 sites in four countries (Spain, France, Italy, United States) between February 27, 2018, and February 17, 2022, and was initially designed to investigate itacitinib in combination with tacrolimus/methotrexate or CsA/MMF for prophylaxis of GVHD in RIC peripheral blood allo-HSCT, with addition of ATG allowed per institutional practice. During the study, the protocol was revised to include itacitinib in combination with PTCy/tacrolimus. Patients were not randomized based on prophylaxis regimen; relative numbers of patients in the various arms reflected treatment preference by site at time of enrollment. When combined with tacrolimus/methotrexate or CsA/MMF, itacitinib was administered orally at 200 mg once daily (qd), starting Day - 3 (3 days before allo-HSCT). For the PTCy/tacrolimus cohort, PTCy 50 mg/kg was given on Days 3 and 4, and itacitinib was started with tacrolimus on Day 5. Patients in all cohorts continued itacitinib 200 mg qd up to Day 90. Itacitinib dose reductions to 100 mg qd and/or interruptions were permitted for toxicity management. At Day 91, itacitinib dose was tapered to 100 mg qd and discontinued at Day 180 unless patients required systemic GVHD treatment, had malignancy relapse or unacceptable toxicity, or withdrew consent (at any earlier time). Immunosuppressant tapering was permitted any time from Day 60 post allo-HSCT, per institutional practice. Infection monitoring, prophylaxis, and treatment were performed per institutional practice. Patients were followed for ≤ 35 days from last dose for safety and up to Day 365 for GVHD, relapse, and survival.

The study was performed in accordance with International Council for Harmonisation Good Clinical Practice, the principles embodied by the Declaration of Helsinki, and local regulatory requirements. The study protocol and all amendments were reviewed and approved by the institutional review board or independent ethics committee of each site before enrollment. All patients provided written informed consent before screening.

### Patients

Eligible patients were aged ≥ 18 years with hematologic malignancy (Table 1). Patients with non-Hodgkin or Hodgkin lymphoma had chemosensitive disease at time of transplant. Other eligibility requirements and exclusion criteria are described in **Supplementary Methods**.

### Endpoints and Assessments

The primary endpoint was hematologic recovery at Day 28, defined as the percentage of patients demonstrating both neutrophil recovery (absolute neutrophil count [ANC] ≥ 500/mm^3^ for three consecutive measurements) and platelet recovery (platelet count ≥ 20 000/mm^3^ with no requirement for platelet transfusion in the preceding 3 days). If ≥ 90% of patients demonstrated hematologic recovery by Day 28, the addition of itacitinib to standard prophylaxis would be considered safe and warrant further study. Secondary endpoints included cumulative incidence of aGVHD (grade 2–4) and cGVHD (mild/moderate/severe); GRFS (percentage of patients who did not experience grade 3–4 aGVHD, cGVHD requiring systemic therapy, malignancy relapse or progression, or death from any cause); relapse-free survival (RFS; time from enrollment to malignancy relapse or progression, or death); overall survival (OS; time from enrollment to death from any cause); transplant-related mortality (percentage of patients who died from causes other than malignancy relapse or disease progression); donor chimerism at Day 28 and during treatment; time to engraftment (neutrophil and platelet recovery); and safety and tolerability, including incidence of treatment-emergent adverse events (TEAEs). TEAEs were tabulated by Standardised Medical Dictionary for Regulatory Activities Queries version 25.0, and severity of TEAEs was assessed using National Cancer Institute Common Terminology Criteria for Adverse Events version 4.03. The relationship of TEAEs to study treatment was based on clinical judgment of study investigators.

The exploratory biomarker analysis is described in **Supplementary Methods**.

### Statistical Analyses

Survival analyses (GRFS, RFS, OS) used the Kaplan-Meier method. Summary statistics were applied to all other efficacy analyses. The efficacy-evaluable population included all patients who received ≥ 1 dose of study treatment and underwent allo-HSCT on Day 0. The safety-evaluable population included all patients who received ≥ 1 dose of study treatment. The pharmacokinetic and translational research-evaluable population included patients who received ≥ 1 dose of study treatment and provided ≥ 1 plasma sample. Determination of sample size is described in **Supplementary Methods**.

## RESULTS

### Patients

**Figure 1:**
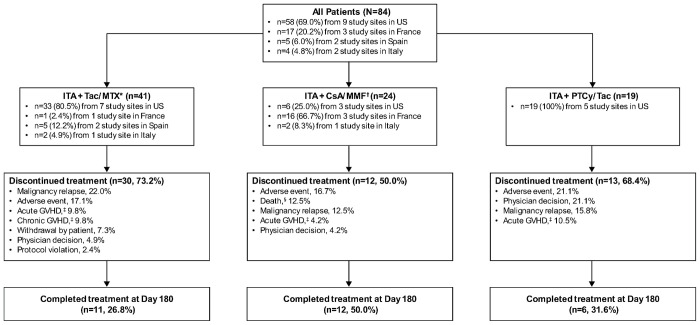
Patient Disposition. Patients received treatment with ITA plus either (1) Tac/MTX (with or without ATG), (2) CsA/MMF (with or without ATG), or (3) PTCy/Tac, depending on institutional practice. ATG, antithymocyte globulin; CsA, cyclosporine A; GVHD, graft-versus-host disease; ITA, itacitinib; MMF, mycophenolate mofetil; MTX, methotrexate; PTCy, post-transplant cyclophosphamide; Tac, tacrolimus. * Treated with ITA + Tac/MTX with ATG (n=9) or without ATG (n=32). ^†^ Treated with ITA + CsA/MMF with ATG (n=16) or without ATG (n=8). ^‡^ Requiring systemic therapy. ^§^ Causes of death were cytomegalovirus infection, post-traumatic intracranial hemorrhage, and sepsis (n=1 patient each).

Eighty-four patients were enrolled (itacitinib + tacrolimus/methotrexate cohort, n = 41; itacitinib + CsA/MMF cohort, n = 24; itacitinib + PTCy/tacrolimus cohort, n = 19). Among all patients, median (range) age was 65.0 (24–76) years; 65.5% had intermediate baseline disease risk (Table 1). Of patients in the itacitinib + tacrolimus/methotrexate cohort, 9/41 (22%) received ATG; 16/24 (66.7%) received ATG with itacitinib + CsA/MMF. Overall, 29 (34.5%) patients completed treatment at Day 180; the most common reasons for early treatment discontinuation were adverse eventss and malignancy relapse (17.9% each; Fig. 1). Of 55 (65.5%) patients with early treatment discontinuation (i.e., before Day 180), 32 discontinued on or after Day 100. Median (range) itacitinib treatment duration was 127.0 (10–189) days, with 70.2% of patients receiving treatment for > 90 days.

### Hematologic Recovery

Fifty-five patients were evaluable for hematologic recovery (i.e., experienced a count nadir below the threshold of 500/mm^3^ for ANC or 20 000/mm^3^ for platelets during the first 28 days after transplant; Table 2). The remaining patients (n = 29) either did not experience a below-threshold ANC count (n = 2) or platelet count (n = 24; of which, 2 received transfusion on or before Day 28) during the first 28 days after transplant, or discontinued the study before Day 28 (n = 4). A total of 51/55 (92.7%) evaluable patients achieved hematologic recovery at Day 28, including 13 (81.3%) who received itacitinib + PTCy/tacrolimus. Three of the four evaluable patients who did not achieve hematologic recovery at Day 28 did so after Day 28 (one patient with Day 34 ANC recovery and Day 28 platelet recovery [secondary myelofibrosis, itacitinib + tacrolimus/methotrexate]; two patients with Day 16 ANC recovery and Day 34 or 38 platelet recovery [itacitinib + PTCy/tacrolimus]). One patient who received itacitinib + PTCy/tacrolimus failed to recover and was diagnosed with primary graft failure followed by relapse. Two patients had secondary graft failure with no evidence of relapse, one on treatment (tacrolimus/methotrexate + ATG; Day 65) and one during post-treatment follow-up (tacrolimus/methotrexate; Day 186); both patients with secondary graft failure underwent second transplantation and were alive at end of study.

Overall, 76 (90.5%) patients achieved full donor chimerism (≥ 95% donor cells) at any time during treatment. At Day 28, 59 (70.2%) patients achieved full donor chimerism and 10 (11.9%) had mixed donor chimerism (5%–94% donor cells). Median (range) time to neutrophil recovery in the total population was 17.0 (4–31) days; median (range) time to platelet recovery was 15.0 (10–41) days. Neutrophil recovery was achieved by 77/78 (98.7%) patients at Day 28, including all 17 (100%) who received itacitinib + PTCy/tacrolimus (Table 2). Platelet recovery was achieved by 53/56 (94.6%) patients at Day 28, including 14 (82.4%) treated with itacitinib + PTCy/tacrolimus (Table 2).

### Efficacy Outcomes

Kaplan-Meier estimates (95% CI) for GRFS in the total population were 60.3% (49.0%–69.9%) at Day 180 and 39.7% (29.2%–50.0%) at Day 365, with median (95% CI) GRFS of 235 (182–356) days (Table 3). Grade 2–4 and grade 3–4 aGVHD cumulative incidences (95% CI) at Day 180 were 13.2% (7.0%–21.5%) and 4.8% (1.5%–10.9%), respectively. Among the 33 patients who experienced aGVHD, 18 (54.5%) had aGVHD with onset during treatment, including two (6%) cases of grade 3–4 aGVHD. For cGVHD, any-grade cumulative incidence (95% CI) in the total population at Day 365 was 37.8% (27.3%–48.3%), and moderate or severe cumulative incidence was 19.9% (11.9%–29.3%). Among the 31 patients who experienced cGVHD, 23 (74.2%) had cGVHD with onset after discontinuation of itacitinib, with a median time from drug discontinuation to cGVHD onset of 62 days (range: 5–310). Kaplan-Meier estimate of OS at 1 year was 78.3% (95% CI: 67.8%–85.7%) for the total population.

**Figure 2:**
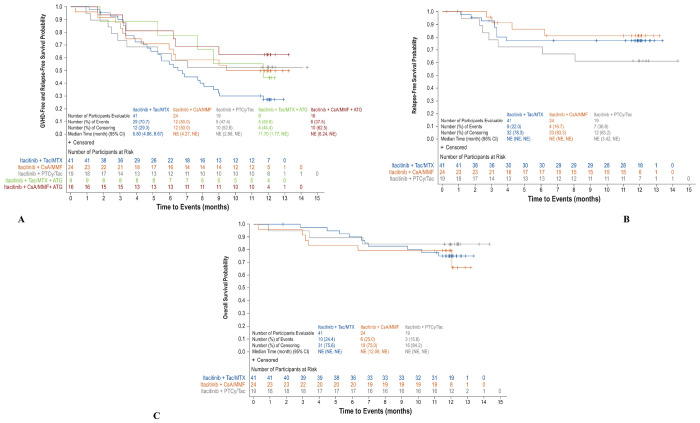
GRFS (A), RFS (B), and OS (C) by Treatment Group. ATG, antithymocyte globulin; GRFS, GVHD-free, relapse-free survival; GVHD, graft-versus-host disease; NE, not evaluable; OS, overall survival; RFS, relapse-free survival.

Kaplan-Meier estimates for GRFS, RFS, and OS by treatment group are shown in Fig. 2. Median (95% CI) GRFS was 6.8 (4.9–8.7) months for itacitinib + tacrolimus/methotrexate; median GRFS was not reached for the other two treatment groups (Fig. 2A). Median RFS and OS were not reached in any groups and appeared to be similar across treatments, although RFS appears to decrease earlier for the itacitinib + PTCy group versus the other two groups (Figs. 2B, 2C).

### Safety

Every patient experienced ≥ 1 TEAE, and 72 (85.7%) had ≥ 1 grade ≥ 3 TEAE (**Table S1**). Anemia and decreased platelet count were the most common any-grade hematologic TEAEs (44.0% each); diarrhea (57.1%) and nausea (51.2%) were the most common nonhematologic TEAEs. Any-grade hematologic events occurred in 59 (70.2%) patients, with grade ≥ 3 hematologic events occurring in 50 (59.5%; **Table S2**).

Overall, 51 (60.7%) patients experienced any-grade itacitinib-related TEAEs, and 31 (36.9%) experienced grade ≥ 3 itacitinib-related TEAEs (**Table S2**). The most common any-grade itacitinib-related TEAEs were platelet count decreased (17.9%) and white blood cell count decreased (15.5%). The most common grade ≥ 3 itacitinib-related TEAEs were platelet count decreased and anemia (9.5% each). Ten (11.9%) patients in the total population had TEAEs leading to itacitinib dose reductions, and 25 (29.8%) had TEAEs leading to itacitinib dose interruption or discontinuation.

Cytopenias as assessed by laboratory counts are presented in **Table S3**. In the itacitinib/PTCy/tacrolimus group, the incidence of grade 3–4 thrombocytopenia (52.6%) was lower than in the itacitinib/tacrolimus/methotrexate (85.4%) and itacitinib/CsA/MMF (83.3%) groups. Similarly, the incidence of grade 3–4 neutropenia was lower in the itacitinib/PTCy/tacrolimus group (31.6%) compared with itacitinib/tacrolimus/methotrexate (95.1%) and itacitinib/CsA/MMF (95.8%).

Serious AEs (SAEs) were experienced by 35 (41.7%) patients overall. The most common SAEs were pyrexia (7.1%), febrile neutropenia (6.0%), and diarrhea (4.8%). Itacitinib-related SAEs were experienced by 11 (13.1%) patients; hepatic cytolysis (n = 2 patients [2.4%]) was the only SAE to occur in ≥ 1 patient.

Grade ≥ 3 infections occurred in 23 (27.4%) patients in the total population, with pneumonia and urinary tract infection the most common (3.6% each; **Table S4**). Clinically notable CMV infection events occurred in 13 patients (15.5%); all except one were CMV reactivation and viremia cases. Four patients (4.8%) died on treatment (ie, within 30 days of last dose of itacitinib; **Table S5**) and 15 patients (17.9%) died during post-treatment follow-up (> 30 days after the last itacitinib dose through Day 371). Additional details relating to infections and deaths are provided in **Supplementary Results**.

### Exploratory Biomarker Analyses

Some changes in biomarker serum concentrations reached statistical significance following itacitinib treatment (**Figure S1**). ST2 concentrations were significantly lower at Day 28 versus baseline in all three analysis cohorts (aGVHD, cGVHD, no GVHD; *P* < 0.05) and remained reduced through Day 100. No significant changes were observed for tumor necrosis factor receptor 1 through Day 100. Interleukin (IL)-6 was significantly increased at Day 28 in patients who developed aGVHD (*P* < 0.05), with numeric increases observed in the other two cohorts. IL-8 levels were unchanged in all three cohorts. Interferon-γ concentrations were significantly higher than baseline at Day 28 for all three groups (all *P* < 0.05) and at later timepoints in patients with aGVHD (Day 56 and 100 [*P* < 0.005 for both timepoints]) and cGVHD (Day 56 [*P* < 0.05]).

## DISCUSSION

In this open-label, proof-of-concept study (GRAVITAS-119), GVHD prophylaxis with itacitinib plus CNI-based regimens or PTCy/tacrolimus was well tolerated, with no detriment to achieving hematologic recovery by Day 28. Cumulative incidence rates for aGVHD in the total population at Day 180 were lower than historical aGVHD rates for patients treated with CNI-based regimens [16, 17]. More than one-third of the total GRAVITAS-119 population developed cGVHD by 1 year, with < 20% of patients developing moderate or severe cGVHD, rates comparable to previously reported data in patients treated with CNI-based regimens [16, 17]. In particular, the group treated with itacitinib and PTCy/tacrolimus had no grade 3–4 aGVHD and a very low rate of moderate or severe cGVHD.

These results compare favorably with those reported for tacrolimus-based, CsA combination, and PTCy-led prophylaxis studies [16, 17]. Recently, the phase 3 BMT CTN 1703 study reported higher 1-year GRFS in patients receiving PTCy/tacrolimus/MMF prophylaxis versus those receiving tacrolimus/methotrexate, establishing PTCy/tacrolimus/MMF as the standard-of-care RIC [15]. The GRFS 1-year estimate for the itacitinib + PTCy/tacrolimus regimen in GRAVITAS-119 was comparable to 1-year GRFS for the PTCy/tacrolimus/MMF regimen in BMT CTN 1703 (52.7%); 1-year OS was also similar across these studies [15]. Further investigation is needed to better understand the potential role for JAK inhibition and the administration of PTCy.

Previously identified biomarkers of aGVHD were evaluated (ST2 and tumor necrosis factor receptor 1), as were cytokines associated with the JAK/signal transducer and activator of transcription (STAT) pathway (IL-6, IL-8, and interferon-γ). We observed a significant and sustained reduction in ST2 levels following transplant and itacitinib combination prophylaxis, suggesting ST2 is a potential pharmacodynamic marker for itacitinib. Although ST2 decrease cannot be solely attributed to itacitinib due to lack of a placebo control arm, ST2 levels were significantly reduced in serum from patients with aGVHD in the itacitinib group but not placebo controls in a previous proteomic biomarker study [24]. Interferon-γ levels were statistically increased by Day 28 in the setting of aGVHD/cGVHD, despite itacitinib combination prophylaxis. Despite significant increases in IL-6 and IL-8 levels in response to itacitinib in a previous study using a murine model of acute inflammation, no changes were observed in IL-6 or IL-8 levels in this study; differences might be attributed to the acute nature of inflammation in the murine model [25]. Although changes in serum biomarkers appeared promising, the magnitude of the overall changes was small. Many biomarker changes in response to itacitinib will require additional validation in larger studies.

Interim results from two phase 2 studies suggest that ruxolitinib prophylaxis is efficacious in preventing GVHD [26]. Although grade 3 – 4 aGVHD rates were low across all treatment regimens in GRAVITAS-119, this did not translate into a corresponding improvement in cGVHD rates, possibly related to stopping itacitinib at Day 180. Despite patients receiving ruxolitinib longer than GRAVITAS-119 patients received itacitinib, the results of the JAK1/JAK2 inhibitor ruxolitinib as a prophylaxis agent suggests that greater JAK1 selectivity does not necessarily translate into better efficacy. It is possible that JAK2 inhibition plays an important role in mitigating acute and chronic inflammation.

In GRAVITAS-119, the most common grade ≥ 3 TEAEs, including treatment-related TEAEs, were cytopenias. Incidence of grade ≥ 3 neutropenia was low with itacitinib/PTCy/tacrolimus (31.6%) relative to other studies of PTCy-based prophylaxis regimens, which have reported grade ≥ 3 neutropenia occurring in 100% of patients [27, 28] and incidence of febrile neutropenia ranging 25%–84% [16, 28–30], albeit with longer follow-up durations than GRAVITAS-119. Infection rates were consistent with those from prior GVHD prophylaxis studies (typically > 50% for CNI-based prophylaxis regimens) [10, 15–17]. Grade ≥ 3 infections occurred in 34.4% of the total population in HOVON-96, similar to the rate in GRAVITAS-119. Rates of CMV reactivation in GRAVITAS-119 also appeared to be consistent with previous studies [10, 16, 17].

Limitations of GRAVITAS-119 include its proof-of-concept, open-label design with relatively small patient numbers in subgroups, limiting cross-group efficacy comparisons. Interpretation of the efficacy of itacitinib in preventing GVHD is limited by the relatively high discontinuation rate and small number of patients. Hematologic recovery at Day 28 was evaluable only in patients whose neutrophil and platelet counts had decreased below required thresholds, and who had not received platelet transfusion in the prior 3 days. Twenty-four patients were not evaluable for hematologic recovery due to their platelet counts not decreasing below 20 000/mm^3^ after transplant; 2 of these had received transfusions. Finally, the exploratory biomarker outcomes require further study before full assessment of itacitinib-based GVHD prophylaxis is possible.

In conclusion, results from GRAVITAS-119 demonstrate that addition of itacitinib as part of GVHD prophylaxis permits timely Day 28 hematologic recovery. Rates of any-grade aGVHD and cGVHD in GRAVITAS-119, as well as the rate of OS, were largely similar to those reported previously in patients receiving CNI-based GVHD prophylaxis regimens; notably, rates of grade 2 – 4 aGVHD appeared to be lower in GRAVITAS-119 [17]. Cytopenias were the most common grade ≥ 3 TEAEs. Addition of itacitinib did not increase infection rates compared with historical data. There remains an unmet need for efficacious and well-tolerated GVHD prophylaxis for patients undergoing allo-HSCT.

## Supplementary Material

This is a list of supplementary files associated with this preprint. Click to download.

- ShahSupplemental.docx
- ShahManuscriptTables.docx

## Figures and Tables

**Table 1:** Patient Demographics and Baseline Clinical Characteristics

Parameter	ITA + Tac/MTX (n = 41)	ITA + CsA/MMF (n = 24)	ITA + PTCy/Tac (n = 19)	All Patients (N = 84)
Age, median (range), y	65.0 (25–76)	64.5 (42–71)	66.0 (24–76)	65.0 (24–76)
< 65 y, n (%)	20 (48.8)	12 (50.0)	7 (36.8)	39 (46.4)
≥ 65 y, n (%)	21 (51.2)	12 (50.0)	12 (63.2)	45 (53.6)
Men, n (%)	22 (53.7)	15 (62.5)	9 (47.4)	46 (54.8)
Race, n (%)				
White	40 (97.6)	10 (41.7)	15 (78.9)	65 (77.4)
Black	1 (2.4)	1 (4.2)	3 (15.8)	5 (6.0)
Other	0	8 (33.3)	1 (5.3)	9 (10.7)
Missing	0	5 (20.8)	0	5 (6.0)
ECOG status score, n (%)				
0	7 (17.1)	15 (62.5)	2 (10.5)	24 (28.6)
1	26 (63.4)	6 (25.0)	15 (78.9)	47 (56.0)
2	8 (19.5)	3 (12.5)	2 (10.5)	13 (15.5)
Underlying malignancy, n (%)				
AML	17 (41.5)	9 (37.5)	4 (21.1)	30 (35.7)
MDS	9 (22.0)	8 (33.3)	7 (36.8)	24 (28.6)
ALL	6 (14.6)	3 (12.5)	1 (5.3)	10 (11.9)
Lymphoma	4 (9.8)	3 (12.5)	7 (36.8)	14 (16.7)
Other	5 (12.2)	1 (4.2)	0 (0.0)	6 (7.1)
Disease status at time of transplant, n (%)				
CR	33 (80.5)	20 (83.3)	11 (57.9)	64 (76.2)
PR	4 (9.8)	0 (0)	3 (15.8)	7 (8.3)
SD	2 (4.9)	3 (12.5)	3 (15.8)	8 (9.5)
Disease risk index, n (%)				
Low	4 (9.8)	3 (12.5)	5 (26.3)	12 (14.3)
Intermediate	27 (65.9)	16 (66.7)	12 (63.2)	55 (65.5)
High	10 (24.4)	5 (20.8)	2 (10.5)	17 (20.2)
HLA donor type, n (%)				
MRD	21 (51.2)	12 (50.0)	3 (15.8)	36 (42.9)
MUD	19 (46.3)	7 (29.2)	12 (63.2)	38 (45.2)
mmURD	1 (2.4)	5 (20.8)	4 (21.1)	10 (11.9)
D/R CMV status, n (%)				
D+/R+	9 (22.0)	9 (37.5)	4 (21.1)	22 (26.2)
D+/R−	6 (14.6)	4 (16.7)	3 (15.8)	13 (15.5)
D−/R+	10 (24.4)	4 (16.7)	6 (31.6)	20 (23.8)
D−/R−	16 (39.0)	7 (29.2)	6 (31.6)	29 (34.5)
Conditioning regimens, n (%)				
Busulfan/fludarabine*	26 (63.4)	7 (29.2)	3 (15.8)	36 (42.9)
Fludarabine/melphalan^†^	10 (24.4)	2 (8.3)	4 (21.1)	16 (19.0)
Busulfan/clofarabine	0	8 (33.3)	0	8 (9.5)
Regimens including TBI	4 (9.8)	3 (12.5)	7 (36.8)	14 (16.7)
Other regimens	1 (2.4)	4 (16.7)	5 (26.3)	10 (11.9)
Received ATG, n (%)	9 (22.0)	16 (66.7)	0	25 (29.8)

ALL, acute lymphocytic leukemia; AML, acute myeloid leukemia; ATG, antithymocyte globulin; CMV, cytomegalovirus; CsA, cyclosporine A; CR, complete response; D/R, donor/recipient; ECOG, Eastern Cooperative Oncology Group; HLA, human leukocyte antigen; ITA, itacitinib; MDS, myelodysplastic syndrome; MMF, mycophenolate mofetil; mmURD, 7/8 mismatched unrelated donor; MRD, 8/8 matched related donor; MTX, methotrexate; MUD, 8/8 matched unrelated donor; PR, partial response; PTCy, post-transplant cyclophosphamide; SD, stable disease; Tac, tacrolimus; TBI, total body irradiation.

* Includes fludarabine phosphate.

† Includes fludarabine phosphate and melphalan hydrochloride.

**Table 2:** Hematologic Recovery at Day 28

Characteristic	Treatment Group			Total (N = 84)
	ITA + Tac/MTX (n = 41)	ITA + CsA/MMF (n = 24)	ITA + PTCy/Tac (n = 19)	
ANC				
Evaluable patients, n*	38	23	17	78
ANC recovery, n (%)	37 (97.4)	23 (100)	17 (100)	77 (98.7)
Time to ANC recovery, days, median (range)	17 (4–31)	18 (11–26)	18 (13–29)	17 (4–31)
Platelets				
Evaluable patients, n*	23	16	17	56
Platelet recovery, n (%)	23 (100)	16 (100)	14 (82.4)	53 (94.6)
Time to platelet recovery, days, median (range)	14 (11–26)	14 (10–18)	24 (16–41)	15 (10–41)
Hematology				
Evaluable patients, n^†^	23	16	16	55
Hematologic recovery, n (%)^‡^	22 (95.7)	16 (100)	13 (81.3)	51 (92.7)

ANC, absolute neutrophil count, CsA, cyclosporine A; ITA, itacitinib; MMF, mycophenolate mofetil; MTX, methotrexate; PTCy, post-transplant cyclophosphamide; Tac, tacrolimus.

* Evaluable patients completed 28 days of follow-up and had ≥ 1 ANC measurement ≤ 500/mm^3^ and/or platelet measurement ≤ 20 000/mm^3^ during the first 28 days after transplant.

† Patients meeting evaluability criteria for both ANC and platelets.

‡ At Day 28, an ANC ≥ 500/mm^3^ for 3 consecutive measurements and a platelet count ≥ 20 000/mm^3^ with no requirement for platelet transfusion in preceding 3 days.

**Table 3:** Efficacy Outcomes (Efficacy-Evaluable Population)

Variable	Treatment Group			Total (N = 84)
	ITA + Tac/MTX (n = 41)	ITA + CsA/MMF (n = 24)	ITA + PTCy/Tac (n = 19)	
GRFS*				
Median GRFS, days (95% CI)	207 (148–264)	NE (130–NE)	NE (87–NE)	235 (182–356)
KM estimate (95% CI) at Day 180	55.1 (38.5–68.9)	66.7 (44.3–81.7)	63.2 (37.9–80.4)	60.3 (49.0–69.9)
With ATG (n = 25)	77.8 (36.5–93.9)	81.3 (52.5–93.5)	N/A	80.0 (58.4–91.1)
Without ATG (n = 59)	48.4 (30.2–64.5)	37.5 (8.7–67.4)	63.2 (37.9–80.4)	51.8 (38.3–63.7)
KM estimate (95% CI) at Day 365	27.3 (14.6–41.6)	50.0 (29.1–67.8)	52.6 (28.7–71.9)	39.7 (29.2–50.0)
With ATG (n = 25)	44.4 (13.6–71.9)	62.5 (34.9–81.1)	N/A	56.0 (34.8–72.7)
Without ATG (n = 59)	22.6 (10.0–38.3)	25.0 (3.7–55.8)	52.6 (28.7–71.9)	32.8 (21.2–44.9)
RFS^†^				
KM estimate (95% CI) at Day 180	77.2 (60.7–87.4)	86.0 (62.5–95.3)	72.2 (45.6–87.4)	78.5 (67.7–86.1)
KM estimate (95% CI) at Day 365	77.2 (60.7–87.4)	81.0 (56.8–92.4)	61.1 (35.3–79.2)	74.6 (63.4–82.8)
TRM				
Patients with TRM, n (%)	6 (14.6)	5 (20.8)	1 (5.3)	12 (14.3)
Cumulative incidence rate at Day 180, % (95% CI)	5.0 (0.9–15.0)	16.7 (5.0–34.1)	5.3 (0.3–22.0)	8.4 (3.7–15.6)
With ATG	NE	18.8 (4.3–41.0)	N/A	12.0 (2.9–28.0)
Without ATG	6.5 (1.1–18.9)	12.5 (0.5–44.5)	5.3 (0.3–22.0)	6.8 (2.2–15.3)
Cumulative incidence rate at Day 365, % (95% CI)	15.1 (6.0–28.0)	20.8 (7.4–39.0)	5.3 (0.3–22.0)	14.5 (7.9–23.0)
With ATG	NE	25.0 (7.3–48.0)	N/A	16.0 (4.9–32.9)
Without ATG	19.4 (7.7–34.9)	12.5 (0.5–44.5)	5.3 (0.3–22.0)	13.8 (6.4–24.0)
aGVHD cumulative incidence rate, % (95% CI)				
Any aGVHD at Day 100	19.5 (9.0–32.9)	25.0 (9.9–43.6)	36.8 (15.9–58.2)	25.0 (16.3–34.7)
With ATG	33.3 (6.7–64.0)	31.3 (10.8–54.5)	N/A	32.0 (14.9–50.6)
Without ATG	15.6 (5.6–30.3)	12.5 (0.5–44.8)	36.8 (15.9–58.2)	22.0 (12.4–33.4)
Any aGVHD at Day 180	31.7 (18.1–46.2)	25.0 (9.9–43.6)	42.1 (19.6–63.2)	32.1 (22.4–42.3)
With ATG	33.3 (6.7–64.0)	31.3 (10.8–54.5)	N/A	32.0 (14.9–50.6)
Without ATG	31.3 (16.1–47.7)	12.5 (0.5–44.8)	42.1 (19.6–63.2)	32.2 (20.7–44.3)
Grade 2–4 at Day 100	7.4 (1.9–18.1)	4.2 (0.3–18.0)	10.5 (1.7–29.1)	7.2 (2.9–14.1)
Grade 2–4 at Day 180	14.9 (5.9–27.7)	8.3 (1.4–23.7)	15.8 (3.7–35.6)	13.2 (7.0–21.5)
Grade 3–4 at Day 100	2.4 (0.2–11.2)	4.2 (0.3–18.0)	0 (NE–NE)	2.4 (0.4–7.5)
Grade 3–4 at Day 180	7.4 (1.9–18.3)	4.2 (0.3–18.0)	0 (NE–NE)	4.8 (1.5–10.9)
cGVHD cumulative incidence rate, % (95% CI)				
Any cGVHD at Day 180	17.5 (7.6–30.8)	16.7 (5.0–34.2)	15.8 (3.7–35.6)	16.9 (9.7–25.7)
With ATG	22.2 (2.8–53.3)	18.8 (4.2–41.2)	N/A	20.0 (7.0–37.7)
Without ATG	16.1 (5.7–31.2)	12.5 (0.5–44.8)	15.8 (3.7–35.6)	15.5 (7.6–26.1)
Any cGVHD at Day 365	46.4 (29.5–61.7)	37.5 (18.4–56.6)	21.1 (6.2–41.7)	37.8 (27.3–48.3)
With ATG	44.4 (11.4–73.9)	43.8 (18.5–66.7)	N/A	45.0 (24.1–63.9)
Without ATG	45.2 (26.9–61.8)	25.0 (2.9–58.3)	21.1 (6.2–41.7)	34.5 (22.5–46.8)
Moderate/severe cGVHD at Day 180	10.0 (3.1–21.7)	0 (NE–NE)	0 (NE–NE)	4.8 (1.6–11.0)
Moderate/severe cGVHD at Day 365	33.9 (19.1–49.3)	8.3 (1.4–23.8)	5.3 (0.3–22.1)	19.9 (11.9–29.3)
KM estimate of OS at 1 y, % (95% CI)^‡^	74.9 (58.4–85.6)	79.2 (57.0–90.8)	84.2 (58.7–94.6)	78.3 (67.8–85.7)

aGVHD, acute graft-versus-host disease; ATG, antithymocyte globulin; cGVHD, chronic graft-versus-host disease; CsA, cyclosporine A; GRFS, GVHD-free, relapse-free survival; ITA, itacitinib; KM, Kaplan-Meier; MMF, mycophenolate mofetil; MTX, methotrexate; N/A, not applicable; NE, not evaluable; OS, overall survival; PTCy, post-transplant cyclophosphamide; RFS, relapse-free survival; Tac, tacrolimus; TRM, transplant-related mortality.

* Number of patients with failure event: ITA + Tac/MTX, 29 (71%); ITA + CsA/MMF, 12 (50%); ITA+PTCy/Tac, 9 (47%); total, 50 (60%). Number of patients censored at last date known alive: ITA + Tac/MTX, 12 (29%); ITA + CsA/MMF, 12 (50%); ITA+PTCy/Tac, 10 (53%); total, 34 (41%).

† Number of censored patients: ITA + Tac/MTX, 32 (78%); ITA + CsA/MMF, 20 (83%); ITA+PTCy/Tac, 12 (63%); total, 64 (76%).

‡ Number of censored patients: ITA + Tac/MTX, 31 (76%); ITA + CsA/MMF, 18 (75%); ITA+PTCy/Tac, 16 (84%); total, 65 (77%).

## Data Availability

Incyte Corporation (Wilmington, DE, USA) is committed to data sharing that advances science and medicine while protecting patient privacy. Qualified external scientific researchers may request anonymized datasets owned by Incyte for the purpose of conducting legitimate scientific research. Researchers may request anonymized datasets from any interventional study (except Phase 1 studies) for which the product and indication have been approved on or after 1 January 2020 in at least one major market (e.g., US, EU, JPN). Data will be available for request after the primary publication or 2 years after the study has ended. Information on Incyte’s clinical trial data sharing policy and instructions for submitting clinical trial data requests are available at: https://www.incyte.com/Portals/0/Assets/Compliance%20and%20Transparency/clinical-trial-data-sharing.pdf?ver=2020-05-21-132838-960
